# Comparative metabolomic analysis of spaghetti meat and wooden breast in broiler chickens: unveiling similarities and dissimilarities

**DOI:** 10.3389/fphys.2024.1456664

**Published:** 2024-10-09

**Authors:** Janghan Choi, Majid Shakeri, Woo Kyun Kim, Byungwhi Kong, Brian Bowker, Hong Zhuang

**Affiliations:** ^1^ USDA-ARS, US National Poultry Research Center, Athens, GA, United States; ^2^ Department of Poultry Science, University of Georgia, Athens, GA, United States

**Keywords:** spaghetti meat, wooden breast, meat quality, metabolomics, NADH, steroid hormones

## Abstract

**Introduction:**

Spaghetti meat (SM) and wooden breast (WB) are emerging myopathies in the breast meat of fast-growing broiler chickens. The purpose of the study was to investigate the metabolomic differences between normal (N), SM, and WB fillets 24 h postmortem.

**Materials and methods:**

Eight chicken breasts for each experimental group were collected from a commercial processing plant. Supernatant from tissue homogenates were subjected to ultra-performance liquid chromatographytandem mass spectrometry (UPLC-MS) analysis.

**Results and methods:**

A total of 3,090 metabolites were identified in the chicken breast meat. The comparison of WB and N showed 850 differential metabolites (*P* < 0.05), and the comparison of SM and N displayed 617 differential metabolites. The comparison of WB and SM showed 568 differential metabolites. The principal component analysis (PCA) plots showed a distinct separation between SM and N and between WB and N except for one sample, but SM and WB were not distinctly separated. Compared to N, 15-Hydroxyeicosatetraenoic acid (15-HETE) increased, and D-inositol-4-phosphate decreased in both SM and WB, indicating that cellular homeostasis and lipid metabolism can be affected in SM and WB. The abundance of nicotinamide adenine dinucleotide (NAD) + hydrogen (H) (NADH) was exclusively decreased between SM and N (*P* < 0.05). Purine metabolism was upregulated in SM and WB compared to N with a greater degree of upregulation in WB than SM. Folic acid levels decreased in SM and WB compared to N (*P* < 0.05). Steroid hormone biosynthesis was downregulated in SM compared to N (*P* < 0.05). Carbon metabolism was downregulated in SM and WB compared to N with greater degree of downregulation in WB than SM (*P* < 0.05). These data suggest both shared and unique metabolic alterations in SM and WB, indicating commonalities and differences in their underlying etiologies and meat quality traits. Dietary supplementation of deficient nutrients, such as NADH, folic acids, etc. and modulation of altered pathways in SM and WB would be strategies to reduce the incidence and severity of SM and WB.

## 1 Introduction

In 2022, broiler meat was the most consumed meat in the US at approximately 45 kg *per capita* (Shahbandeh, 2023). The fast growth and high feed efficiency traits of broiler chickens, achieved through advancements in genetic selection and nutritional programs, have significantly contributed to making broiler meat an affordable and nutritious protein source for human consumption (Choi et al., 2023). However, it is critical to acknowledge that muscle myopathies including spaghetti meat (SM), wooden breast (WB), and white stripping (WS) are frequently observed in the breast meat of fast-growing broiler chickens (Che et al., 2022). The incidence of WB is estimated to be 5%–10% in the commercial condition (Baltic et al., 2024) and could be greater than 50% in certain cases (Xing et al., 2020). The incidence of SM could be up to 20% in certain cases (Baldi et al., 2021).

A number of studies investigated the potential etiologies and meat quality traits of WB, characterized by tough texture and high-water content, and of WS, characterized by the development of white striations parallel to the orientation of the myofibers (Kuttappan et al., 2012; Velleman and Clark, 2015; Abasht et al., 2016; Choi et al., 2024; Bailey et al., 2015) suggested that WB and WS may have similarities in their etiologies because of their macroscopic and microscopic similarities. Although diverse omics analyses including transcriptomic, proteomics, metabolomics, and lipidomics were performed in WB and WS (Kong et al., 2021; Kang et al., 2022; Liu et al., 2022; Wang et al., 2023; Kong et al., 2024), there are still no clear explanations for exact etiologies to date for WB. SM, described by soft texture and loss of muscle integrity due to the detachment of muscle fibers, is another emerging muscle myopathy in broilers (Baldi et al., 2021). Not many studies have been conducted on SM due to its recentness (Baldi et al., 2021; Che et al., 2024) demonstrated that SM exhibited inflammation and fibrosis similar to those observed in WB, both of which are common traits of muscle myopathies. However, etiologies and quality characteristics of SM are also not fully understood. Understanding the similarities and dissimilarities between SM and WB would be instrumental in finding the etiologies of these myopathies and potential solutions to reduce their incidence and severity in broiler chickens. A recent study by Che et al. (2024) investigated the transcriptomic differences between SM, WB, and N and demonstrated that SM may have similar etiologies with WB at the transcriptomic level. However, to our best knowledge, there are no published studies that compared SM and WB at the metabolomic level. Therefore, the purpose of the study was to investigate the metabolomic differences between SM, WB, and N at 24 h *postmortem*.

## 2 Materials and methods

### 2.1 Sample collection

Eight fillets (pectoralis major) for each experimental groups, N without any muscle myopathies, severe WB [score 3 according to Bowker et al. (2019)], and severe SM [score 2 according to Baldi et al. (2021)] were selected from a batch of broiler breast meat collected from a commercial slaughterhouse. Samples (size 1 cm × 1 cm × 1 cm) were excised from the cranial-ventral portion of the muscle at 24 h *postmortem* and stored at −80°C until further analysis. For metabolomics analysis, 100 mg of the frozen samples were placed in 2 mL tubes with liquid nitrogen and shipped with dry ice to a commercial company (Metware Biotechnology, Woburn, MA).

### 2.2 Untargeted metabolomic analysis

Samples were homogenized in a ball-mill grinder at 30 Hz (Hz) for 20 s. 400 μL solution (Methanol:Water = 7:3, V/V) containing internal standard was mixed with 20 mg of ground sample and mixed in a shaker at 2,500 rpm for 5 min. The mixture was placed on ice for 15 min and centrifuged at 12,000 rpm for 10 min at 4°C (5430R; Eppendorf, Hamburg, Germany). The 300 μL of the supernatant was collected and placed in −20°C for 30 min. The sample was then centrifuged at 12,000 rpm for 3 min at 4°C, and the supernatant was collected for the untargeted metabolomic analysis. Ultra-performance liquid chromatography (ExionLC; SCIEX, CA)-tandem mass spectrometry (TripleTOF 6,600+; SCIEX, CA) (UPLC-MS) equipped with Acquity UPLC HSS T3 (1.8 μm, 2.1 mm × 100 mm) column (Waters, Milford, MA) was used for untargeted metabolomics. The column temperature was 40°C, and ultrapure water and acetonitrile were mobile phase A and B, respectively. Both mobile phases contained 0.1% formic acid. The flow rate was 0.4 mL/min, and the injection volume was 2 μL.

### 2.3 Quality control

A quality control (QC) sample was prepared from a mixture of all sample extracts to examine the reproducibility of the entire metabolomics process. During data collection, one quality control sample was inserted for every 10 test samples.

### 2.4 Data processing, multivariate, and enrichment analyses

The original data file acquired by UPLC-MS was converted to mzXML format by ProteoWizard. Peak extraction, peak alignment and retention time correction were performed by XCMS program. The peaks with missing rate >50% in each group of samples were filtered. The blank values were filled with k-nearest neighbors (KNN), and the peak area was corrected by the support vector regression (SVR) method. The metabolites were annotated by searching the MetwareBio’s in-house database, integrated public database, prediction database and metDNA. Finally, substances with a comprehensive identification score above 0.7 and a coefficient of variation (CV) value of QC samples less than 0.3 were extracted, and then positive and negative mode were combined (substances with the greatest qualitative grade and the lowest CV value were retained) to obtain the ALL_sample_data file.

Principal component analysis (PCA) was calculated by using the base package of R software (version 4.1.2) with parameter scale = True indicating unit variance Scaling (UV) for normalizing the data. Discriminant analysis by orthogonal partial least squares (OPLS-DA) was analyzed by using MetaboAnalystR of R software (version 4.1.2). R^2^Y (goodness of fit) was higher than 0.99 in the comparison between SM and N, SM and WB, and WB and N. Cumulative Q^2^ (goodness of prediction) was 0.844, 0.667, and 0.690 in the comparison between SM and N, SM and WB, and WB and N, respectively. Q^2^ > 0.5 can be considered as an effective model, and Q^2^ > 0.9 can be considered as an excellent model. OPLS-DA S-plots were generated by setting variable importance in projection (VIP) value >1 as significant. The horizontal axis is the covariance between the principal components and metabolites, the vertical axis represents the correlation coefficient between the principal components and the metabolites. The closer the points are to the top right corner or bottom left corner, the more significant the difference in metabolite abundance. Red dots indicate metabolites with VIP value >1 and green dots indicate metabolites with VIP value ≤1. Volcano plots were generated with VIP, log_2_ fold changes, and -log_10_ *P*-value by using R software. Hierarchical clustering trees were generated according to the relative intensity of the differential metabolites by using R software. Differential metabolites were screened by using the following condition: VIP >1 and *P* < 0.05. The top 20 differentially expressed metabolites regardless of up- and downregulation were selected by using the values of log_2_ fold change between N, SM, and WB. The top 10 up- and downregulated differentially expressed metabolites were also screened by using the values of log_2_ fold change between N, SM, and WB. To identify metabolites with significant alterations unique to SM and WB, as well as those shared between them compared to N, the product of the log2 fold changes between SM and N and between WB and N was calculated. Uniquely differential metabolites in SM or WB with high fold changes (the top 10 up- and downregulated) were selected by identifying the screened metabolites (*P* < 0.05) in one group but not screened (*P* > 0.05) in the other group. For enrichment analysis, differential metabolites (*P* < 0.05) were annotated using the KEGG database (Kanehisa and Goto, 2000).

## 3 Results

### 3.1 Number of the differential metabolites

**FIGURE 1:**
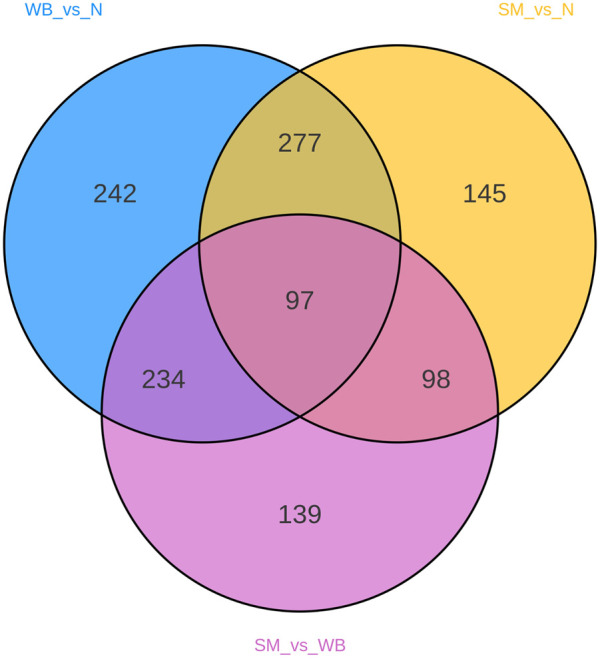
Venn diagram of differential metabolites between spaghetti meat (SM), wooden breast (WB), and normal (N) fillets.

A total of 3,090 metabolites were identified in the chicken breast meat. Table 1 showed the number of differential metabolites between the experimental groups. The comparison of SM and N, SM and WB, and WB and N showed 617-, 568-, and 850 differential metabolites, respectively. The Venn diagram shows the number of shared and unique differential metabolites in the different comparisons (Figure 1).

**TABLE 1:** Number of differential metabolites with statistical significance (*P* < 0.05) in the comparisons of spaghetti meat (SM) and normal (N) fillets, spaghetti meat (SM) and wooden breast (WB), and spaghetti meat (SM) and wooden breast (WB).

Comparisons	Total	Downregulated	Upregulated
SM and N	617	342	275
SM and WB	568	314	254
WB and N	850	362	488

### 3.2 PCA and OPLS-DA plots

**FIGURE 2:**
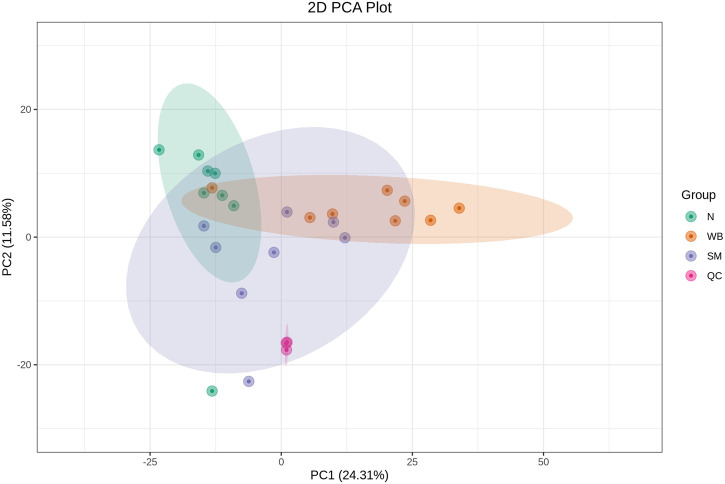
Principal component analysis (PCA) score diagrams of different experimental groups including spaghetti meat (SM), wooden breast (WB), and normal (N) with quality control (QC) samples.

**FIGURE 3:**
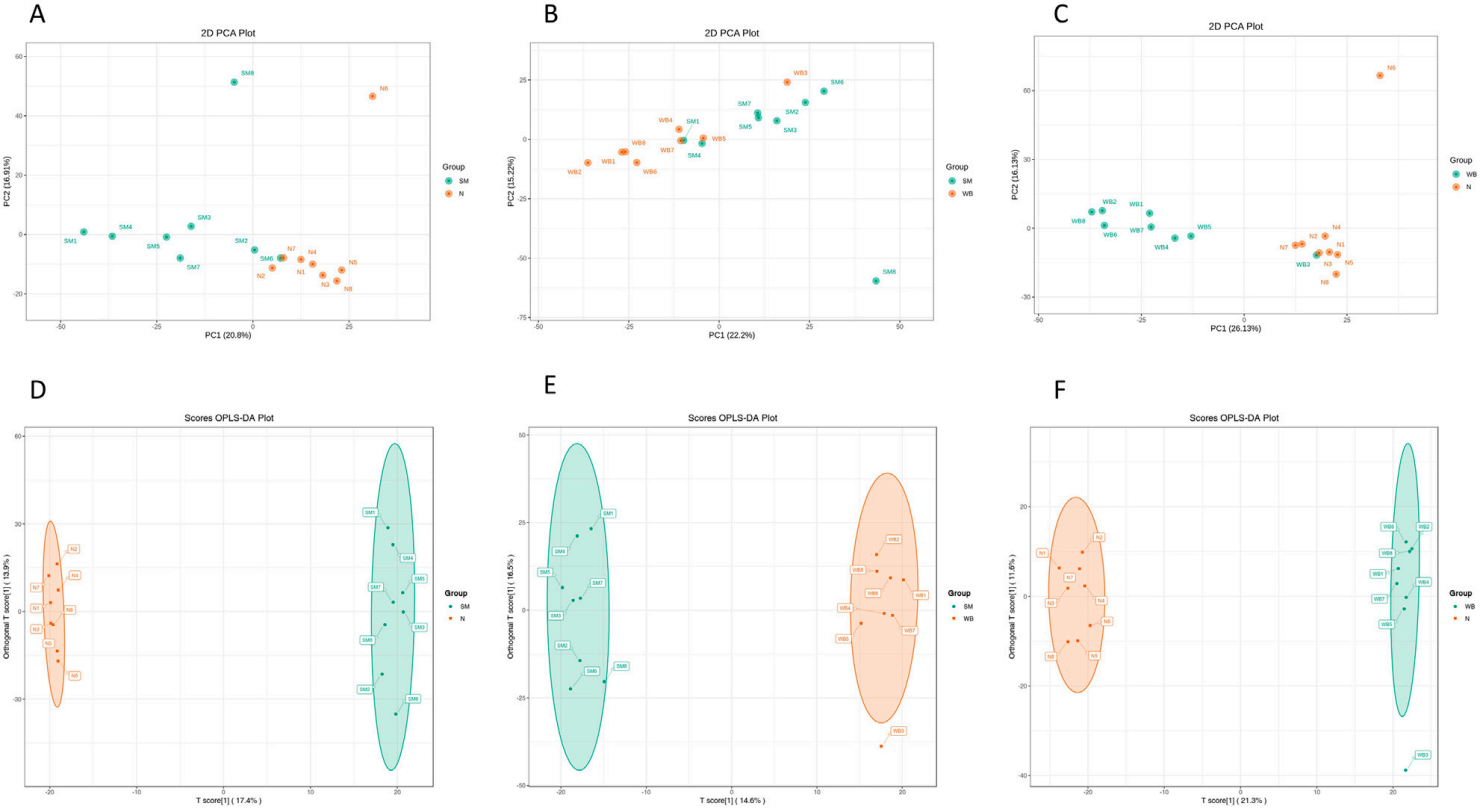
Principal component analysis PCA; **(A–C)** and discriminant analysis by orthogonal partial least squares [(OPLS-DA; **(D, E, and F**)] of spaghetti meat (SM), wooden breast (WB), and normal (N) groups based on differential metabolites when wooden breast (WB) or spaghetti meat (SM) compared to normal (N) fillets and each other. A and D: SM and N, B and E: SM and WB, C and F: WB and N.

**FIGURE 4:**
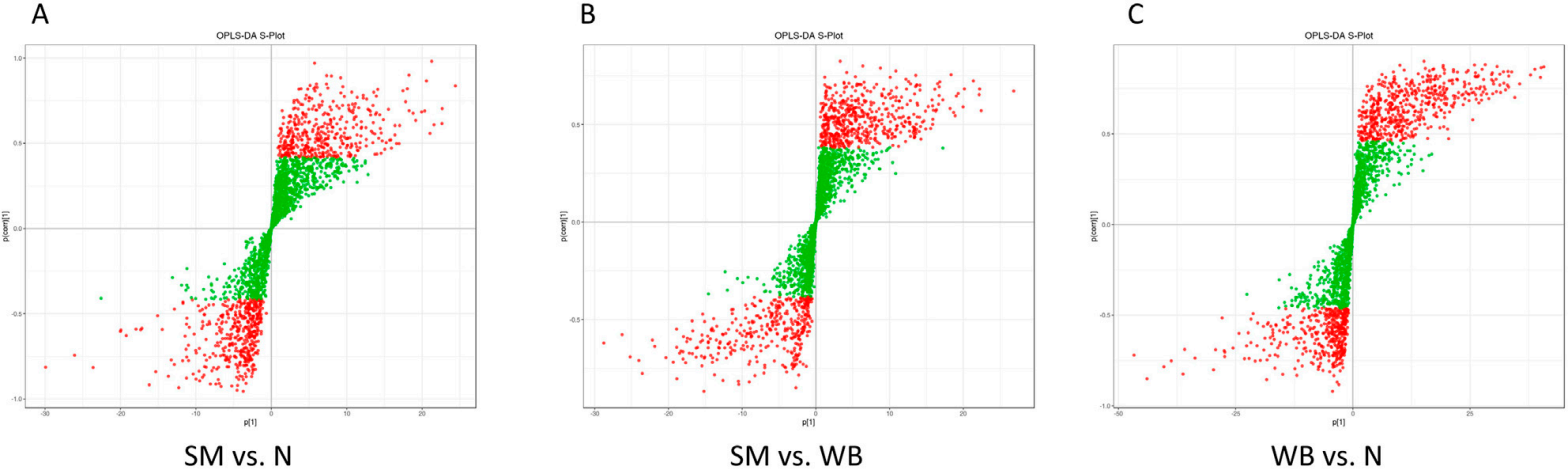
Discriminant analysis by orthogonal partial least squares (OPLS-DA) S-plot of spaghetti meat (SM), wooden breast (WB), and normal (N) groups. **(A)** SM and N; **(B)** SM and WB; and **(C)** WB and N. The *X*-axis represents the covariance between the principal components and metabolites, the *Y*-axis represents the correlation coefficient between the principal components and the metabolites. The closer the points are to the top right corner or bottom left corner, the more significant the difference in metabolite abundance. Red dots indicate metabolites with variable importance in projection (VIP) value >1 and green dots indicate metabolites with VIP value ≤1.

As shown in Figure 2, the QC samples were tightly clustered in a specific area, which indicates the variations among the samples are attributed to biological differences. The PCA plots showed distinct separation between SM and N and between WB and N except for one sample and have PC1 greater than 20% and PC2 greater than 15% in the comparisons (Figures 3A, C). However, SM and WB were not distinctly separated in the PCA plot (Figure 3B). The OPLS-DA plots displayed distinct separation between SM and N, SM and WB, and WB and N (Figures 3D–F). The OPLS-DA S-plots (Figure 4) indicated that numerous metabolites contributed to separating the comparison groups. The comparison of SM and WB seemed more clustered near the *Y*-axis, which indicated that there were less significant metabolites for the separation.

### 3.3 Volcano plots of differential metabolites and hierarchical clustering trees

**FIGURE 5:**
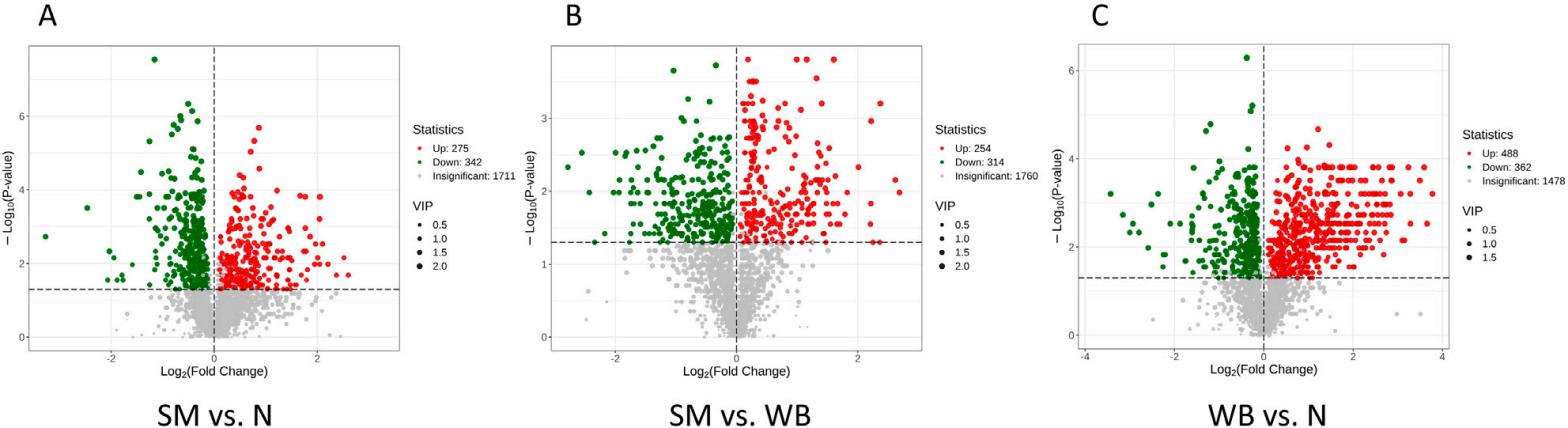
Volcano plots of differential metabolites when spaghetti meat (SM) or wooden breast (WB) compared to normal (N) fillets and each other. A: SM and N; B: SM and WB; and C: WB and N. Each point in the volcano plot represents a metabolite with green points representing downregulated differential metabolite, red points upregulated differential metabolite, and gray points the detected metabolites but show no significant differences. The *X*-axis represents the log_2_ fold change value of metabolites between two groups, and the Y-axis represent the level of significant differences (-log10 *P*-value). The size of each dot represents the variable importance in projection (VIP) value.

**FIGURE 6:**
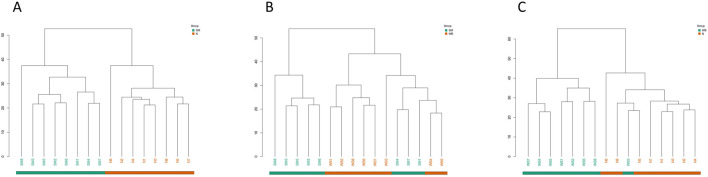
Hierarchical clustering trees of differential metabolites when spaghetti meat (SM) or wooden breast (WB) compared to normal (N) fillets and each other. **(A)** SM and N; **(B)** SM and WB; and **(C)** WB and N.

The volcano plots of the comparisons between N, SM, and WB are shown in Figure 5. The comparisons of WB and N and of SM and WB showed the greatest- and the lowest number of differential metabolites, respectively. The hierarchical clustering trees of the comparisons of SM and N, SM and WB, and WB and N were shown in Figure 6. The comparisons of SM and N and of WB and N showed that either SM or WB were distinctly separated compared to the N, but the comparison of SM and WB did not show distinct separation.

### 3.4 Differential metabolites

Differential metabolites between N, SM, and WB are presented in Supplementary Table 1. Table 2 displays the top 10 up- and downregulated metabolites between N, SM, and WB. Folic acid was observed in the top 10 downregulated differential metabolites in comparisons between SM and N and between WB and N. Neoxanthin was observed in the top 10 downregulated differential metabolites in comparisons between WB and N. Taurine, glutathione, and reduced glutathione were upregulated in SM compared to N and WB compared to N (*P* < 0.05). In the comparisons between SM and WB and between WB and N, 13 and 10 differential metabolites, respectively, were amino acid related metabolites among the top 10 upregulated or downregulated metabolites. There were only 6 amino acid related metabolites (amino acids, polypeptides, small peptides, and amino acid derivatives) among the top 10 upregulated or downregulated metabolites between SM and N. Several differential metabolites associated with amino acid related metabolites were observed in SM (185) and WB (312) when compared to N. Between SM and N, 103 amino acid related metabolites were downregulated, but 82 amino acid related metabolites were upregulated. Between WB and N, 111 amino acid related metabolites were downregulated, but 201 amino acid related metabolites were upregulated. Between SM and WB, there were 211 amino acid related differential metabolites (up: 86 and down: 125) were associated with amino acid related metabolites.

**TABLE 2:** The top 10 upregulated or downregulated differential metabolites when spaghetti meat (SM) or wooden breast (WB) compared to normal (N) fillets and each other.

Comparison	Up- or downregulated	Order	Compounds	VIP	*p*-value	Log_2_FC
SM vs. N	Up	1	(±)15-HETE	1.476	0.021	2.601
		2	Phe-Tyr-Phe-Lys-Ile	1.315	0.007	2.517
		3	alpha-Dimorphecolic acid	1.457	0.021	2.371
		4	NCGC00386087-01_C43H60N2O12_(2S)-N-[(2E,4E,6S,7R)-7-{(3S,4R)-3,4-Dihydroxy-5-[(1E,3E,5E)-7-(4-hydroxy-2-oxo-1,2-dihydro-3-pyridinyl)-6-methyl-7-oxo-1,3,5-heptatrien-1-yl]tetrahydro-2-furanyl}-6-methoxy-5-methyl-2,4-octadien-1-yl]-2-{(2R,3R,4R,6S)-2,3,4-trihydroxy-5,5-dimethyl-6-[(1E,3E)-1,3-pentadien-1-yl]tetrahydro-2H-pyran-2-yl}butanamide	1.685	0.010	2.207
		5	N-Acetyl-L-aspartic acid	1.654	0.003	2.102
		6	Indoleacetaldehyde	2.351	<0.001	2.051
		7	15(S)-Hydroxyeicosatrienoic acid	1.519	0.010	2.046
		8	1-Palmitoyl-2-oleoyl-sn-glycero-3-phosphocholine	2.076	0.001	2.043
		9	Asn-Gly-Glu-Val-Lys	1.619	0.007	2.007
		10	Lys-Met-Val-Ser-Arg	1.652	0.003	1.966
	Down	1	Stigmastane-3,6-dione	1.779	0.002	−3.271
		2	NADH	1.953	<0.001	−2.461
		3	methyl 4-(6-(4-(methoxycarbonylamino)phenyl)-4-morpholino-1H-pyrazolo [3,4-d]pyrimidin-1-yl)piperidine-1-carboxylate	1.425	0.028	−2.065
		4	PE-NMe(22:6 (4Z,7Z,10Z,13Z,16Z,19Z)/18:1 (9Z))	1.948	0.005	−2.032
		5	D-inositol-4-phosphate	1.422	0.007	−1.946
		6	Ala-Lys	1.413	0.028	−1.883
		7	6-o-Phosphonohexopyranose	1.444	0.021	−1.789
		8	beta-Alanyl-L-lysine	1.400	0.028	−1.769
		9	Folic acid	1.226	0.011	−1.587
		10	Furathiocarb	2.196	<0.001	−1.499
SM vs. WB	Up	1	Leucylvaline	1.805	0.010	2.687
		2	Leu-Val	1.856	0.007	2.618
		3	Valylcysteine	2.027	0.001	2.372
		4	Cycluron	1.433	0.050	2.362
		5	His-Phe-Tyr-Asp	1.509	0.050	2.248
		6	Carnitine C10:0	2.099	0.001	2.220
		7	2-[(2-Amino-3-phenylpropanoyl)amino]-3-methylbutanoic acid	1.816	0.015	2.215
		8	Val-val	1.666	0.028	2.208
		9	L-Octanoylcarnitine	1.885	0.005	2.010
		10	Phe-Glu	1.676	0.010	1.825
	Down	1	Lys-Thr-His	1.757	0.005	−2.781
		2	Val-Leu-Ser-Pro-Ala	1.894	0.003	−2.552
		3	N-tridecanoyl-L-Homserine lactone	1.708	0.010	−2.428
		4	PA (18:0/18:3 (9Z,12Z,15Z))	1.493	0.049	−2.341
		5	Maytansine	1.545	0.038	−2.174
		6	Tyr-Leu-Ala-Lys	1.785	0.010	−2.029
		7	NADH	1.880	0.003	−1.995
		8	Tyr-Pro-His	1.724	0.010	−1.939
		9	N-Acetylserotonin	1.784	0.007	−1.931
		10	Tyr-Phe-Lys	1.689	0.003	−1.838
WB vs. N	Up	1	Lys-Thr-Glu	1.758	0.001	3.775
		2	Lys-Thr-His	1.682	0.003	3.657
		3	(±)15-HETE	1.886	<0.001	3.590
		4	alpha-Dimorphecolic acid	1.874	<0.001	3.493
		5	Pro-Gln-Lys	1.643	0.003	3.283
		6	Tyr-Pro-His	1.787	0.001	3.279
		7	15-Hydroxy-5,8,11,13-eicosatetraenoic acid	1.857	<0.001	3.237
		8	Leu-Thr-Pro-Asp-Ala	1.610	0.007	3.149
		9	D-myo-Inositol-1,3,4,5,6-pentaphosphate	1.692	0.007	3.102
		10	Ala-Pro-Asp-Ala-Lys	1.825	0.001	3.066
	Down	1	D-inositol-4-phosphate	1.785	0.001	−3.429
		2	Ala-Lys	1.698	0.002	−3.154
		3	Stigmastane-3,6-dione	1.473	0.005	−2.999
		4	beta-Alanyl-L-lysine	1.628	0.003	−2.928
		5	Folic acid	1.596	0.005	−2.793
		6	(2RS)-Lotaustralin	1.559	0.010	−2.587
		7	His-Phe-Tyr-Asp	1.842	0.001	−2.514
		8	Valylcysteine	1.736	0.001	−2.366
		9	Neoxanthin	1.292	0.028	−2.256
		10	Clopidogrel	1.490	0.015	−2.245

VIP, variable importance in the projection; FC, fold change; (±) 15-HETE, 15-Hydroxyeicosatetraenoic acid; NADH, Nicotinamide adenine dinucleotide.

### 3.5 Co-expressed and uniquely expressed metabolites in SM and WB

Co-expressed and uniquely expressed metabolites in SM and WB compared to N are presented in Supplementary Table 2. Between SM and N, 140 out of 617 differential metabolites were co-expressed with those from the comparison of WB and N, and 477 metabolites were uniquely expressed in both WB and SM. Between WB and N, 373 out of 850 differential metabolites were co-expressed with the screened metabolites from the comparison of SM and N. The top 10 co-expressed metabolites between SM and WB are shown in Table 3. 15-hydroxyeicosatetraenoic acid (15-HETE) increased and D-inositol-4-phosphate decreased with high fold changes in both SM and WB compared to N (*P* < 0.05). Several lipid related metabolites including alpha-dimorphecolic acid, 15(S)-HETE, and (±) 18-HEPE were upregulated in SM and WB compared to N. Stigmastane-3,6-dione was the most decreased differential metabolites in SM and WB compared to N. In addition, Ala-Lys and beta-Alanyl-L-lysine were one of the most decreased differential metabolites in SM and WB compared to N. There were no metabolites showing opposite direction of fold change (e.g., minus values after multiplication of fold changes) between the SM and WB compared to N. The top 10 uniquely expressed in SM or WB compared to N are shown in Table 4. The abundance of nicotinamide adenine dinucleotide (NAD) + hydrogen (H) (NADH) was exclusively decreased in SM compared with N (*P* < 0.05). However, no significant differences were observed between WB and N (*P* > 0.05).

**TABLE 3:** The top 10 co-expressed differential metabolites and their fold changes (FC) and multiplied values in spaghetti meat (SM) and wooden breast (WB) compared to normal (N) fillets.

	Metabolites	SM and N Log_2_FC	WB and N Log_2_FC	Multiplied value
1	Stigmastane-3,6-dione	−3.271	−2.999	9.810
2	(±)15-Hydroxyeicosatetraenoic acid (HETE)	2.601	3.590	9.337
3	alpha-Dimorphecolic acid	2.371	3.493	8.282
4	D-inositol-4-phosphate	−1.946	−3.429	6.672
5	Phe-Tyr-Phe-Lys-Ile	2.517	2.534	6.379
6	Ala-Lys	−1.883	−3.154	5.937
7	N-Acetyl-L-aspartic acid	2.102	2.814	5.915
8	Leu-Thr-Pro-Asp-Ala	1.810	3.149	5.699
9	15(S)-Hydroxyeicosatrienoic acid	2.046	2.714	5.553
10	beta-Alanyl-L-lysine	−1.769	−2.928	5.179

**TABLE 4:** The top 10 uniquely expressed metabolites between spaghetti meat (SM) or wooden breast (WB) compared to normal (N) fillets.

Unique differential metabolites between SM and N			
Metabolites	VIP	*p*-value	Log_2_FC
NADH	1.953	<0.001	−2.461
NCGC00386087-01_C43H60N2O12_(2S)-N-[(2E,4E,6S,7R)-7-[(3S,4R)-3,4-Dihydroxy-5-[(1E,3E,5E)-7-(4-hydroxy-2-oxo-1,2-dihydro-3-pyridinyl)-6-methyl-7-oxo-1,3,5-heptatrien-1-yl]tetrahydro-2-furanyl]-6-methoxy-5-methyl-2,4-octadien-1-yl]-2-[(2R,3R,4R,6S)-2,3,4-trihydroxy-5,5-dimethyl-6-[(1E,3E)-1,3-pentadien-1-yl]tetrahydro-2H-pyran-2-yl]butanamide	1.685	0.010	2.207
PE-NMe(22:6 (4Z,7Z,10Z,13Z,16Z,19Z)/18:1 (9Z))	1.948	0.005	−2.032
Asn-Gly-Glu-Val-Lys	1.619	0.007	2.007
Etoposide	2.006	0.002	1.859
Lyso-PAF C-16-d4	1.782	0.005	1.779
Ile-Ile	1.937	0.001	1.768
CDP-ethanolamine	1.487	0.015	1.462
(2S,4S)-1-Acetoxy-16-heptadecene-2,4-diol	1.637	0.001	−1.258
Aspartylthreonine	1.235	0.050	1.221
Unique differential metabolites between WB and N			
Lys-Thr-Glu	1.758	0.001	3.775
Lys-Thr-His	1.682	0.003	3.657
15-Hydroxy-5,8,11,13-eicosatetraenoic acid	1.857	<0.001	3.237
D-myo-Inositol-1,3,4,5,6-pentaphosphate	1.692	0.007	3.102
13-HOTE	1.776	<0.001	2.873
N-Acetylserotonin	1.831	<0.001	2.865
Phe-Lys-Tyr	1.826	<0.001	2.855
Pro-Ile-Thr	1.668	0.001	2.825
Phe-Val-Ser	1.646	0.002	2.755
3,5-Pyridinedicarboxylic acid, 1,4-dihydro-2,4,6-trimethyl-, diethyl ester	1.520	0.005	2.743

VIP, variable importance in the projection; FC, fold change; NADH, nicotinamide adenine dinucleotide.

### 3.6 Kyoto encyclopedia of genes and genomes (KEGG)-enriched analysis of differentiated metabolites

**FIGURE 7:**
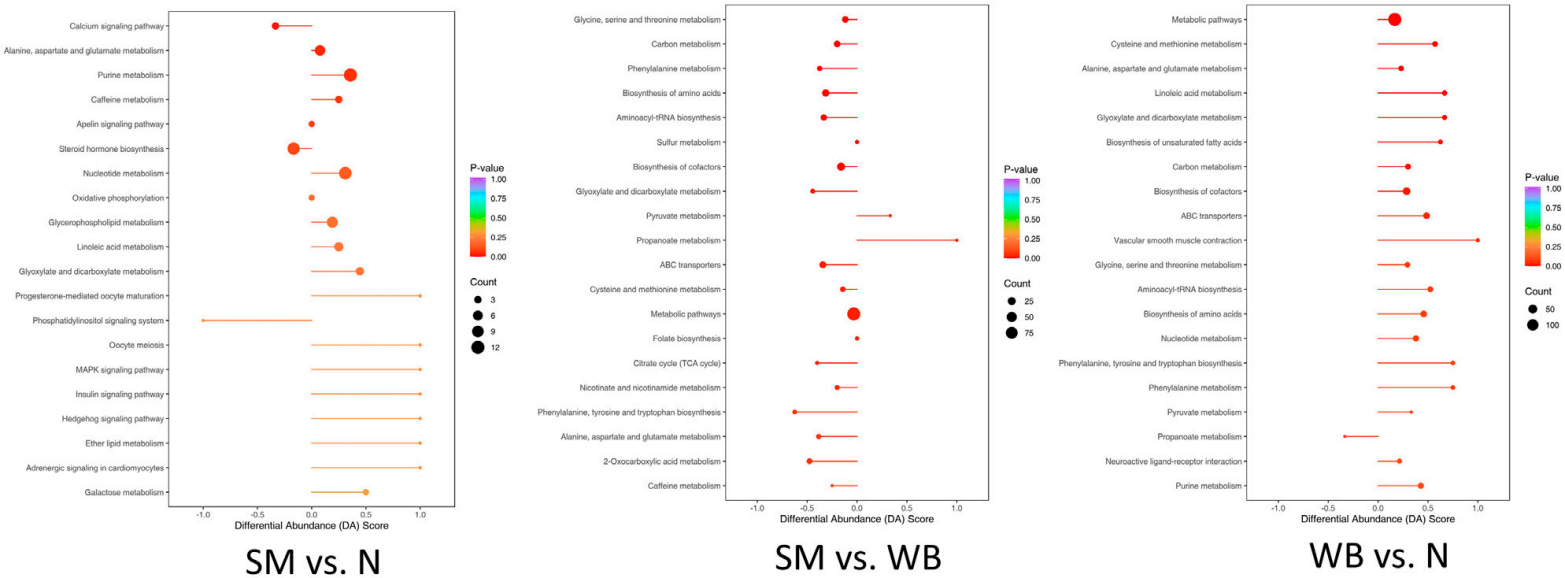
Top 20 Kyoto Encyclopedia of Genes and Genomes (KEGG) enriched pathways with *P*-value and differential abundance (DA) score. DA score was calculated as follows: number of upregulated differential metabolites annotated to a pathway–number of downregulated differential metabolites annotated to the pathway)/total number of metabolites annotated to the pathway.

The top 10 differentiated Kyoto Encyclopedia of Genes and Genomes (KEGG) pathways between N, SM, and WB are presented in Table 5. The top 20 upregulated or downregulated KEGG pathways between N, SM, and WB are shown in Figure 7. Metabolic pathways and biosynthesis of cofactors were the two most annotated KEGG pathways between N, SM, and WB. There were 10 and 5 differential metabolites that were related to steroid hormone biosynthesis between SM and N and between WB and N, respectively. The 7 and 3 differential metabolites related to steroid hormone biosynthesis were downregulated and upregulated, respectively, between SM and N. 21-deoxycortisol, aldosterone hemiacetal, tetrahydrodeoxycorticosterone, androsterone, cortisone, 11 beta-hydroxyandrostenedione, and (8S,9S,10S,13S,14S)-17-(2-hydroxyacetyl)-10,13-dimethyl-2,4,5,6,7,8,9,12,14,15,16,17-dodecahydro-1H-cyclopenta [a] phenanthrene-3,11-dione, were downregulated, testosterone, 16 alpha-hydroxyestrone, and 2-methoxyestradiol were upregulated in SM compared to N. There were 21, 10, and 16 differential metabolites related to ATP-binding cassette (ABC) transporters; 20, 10, and 19 related to biosynthesis of amino acids; 13, 3, and 13 related to aminoacyl-tRNA biosynthesis; 14, 5, and 14 related to carbon metabolism; and 16, 12, and 11 related to purine metabolism between WB and N, SM and N, and SM and WB, respectively. Between SM and WB, 8 differential metabolites related to purine metabolism biosynthesis were downregulated and 3 upregulated.

**TABLE 5:** Top 10 differentiated Kyoto Encyclopedia of Genes and Genomes (KEGG) pathways based on number of significant differential metabolites between spaghetti meat (SM), wooden breast (WB), and normal (N) fillets.

SM and N		SM and WB		WB and N	
KEGG pathway	Number of differential metabolites	KEGG pathway	Number of differential metabolites	KEGG pathway	Number of differential metabolites
Metabolic pathways	87	Metabolic pathways	98	Metabolic pathways	147
Biosynthesis of cofactors	17	Biosynthesis of cofactors	25	Biosynthesis of cofactors	32
Purine metabolism	12	Biosynthesis of amino acids	19	ATP-binding cassette (ABC) transporters	21
Nucleotide metabolism	11	ATP-binding cassette (ABC) transporters	16	Biosynthesis of amino acids	20
ABC transporters	10	Carbon metabolism	14	Nucleotide metabolism	17
Steroid hormone biosynthesis	10	Glycine, serine and threonine metabolism	14	Purine metabolism	16
Glycerophospholipid metabolism	8	Aminoacyl-tRNA biosynthesis	13	Carbon metabolism	14
Alanine, aspartate and glutamate metabolism	7	Nucleotide metabolism	11	Aminoacyl-tRNA biosynthesis	13
Pyrimidine metabolism	5	Purine metabolism	11	Cysteine and methionine metabolism	12
Neuroactive ligand-receptor interaction	5	2-Oxocarboxylic acid metabolism	10	Glycerophospholipid metabolism	11

## 4 Discussion

The purpose of the study was to investigate the metabolomic differences between N (non-myopathic), SM, and WB meat at 24 h *postmortem*. The research interests were: 1) to identify similar metabolites expressed in SM and WB and 2) to identify unique metabolites expressed exclusively in SM or WB. These findings are instrumental in understanding the similarities and differences in the etiologies and meat quality traits of SM and WB in broiler fillets. In the current study, the 24 h *postmortem* time point was selected because we aimed to compare metabolomic differences between the groups after the completion of rigor mortis and other changes that take place in the tissue during the early *postmortem* conversion of muscle to meat. PCA and OPLS-DA plots showed that SM or WB was distinctly separated compared to N, which indicates that SM or WB retains different metabolic state compared to N. However, SM and WB samples were not distinctly separated when compared each other according to the PCA plot. Furthermore, hierarchical clustering tree analysis also showed consistent trends. These results may indicate that there are common etiologies or traits in meat quality in SM and WB. A recent transcriptomics study by Che et al. (2024), which had a similar experimental design to the current study, reported that some traits of SM and WB were similar but the other dissimilar at the transcriptomic level of SM and WB. Our current results also indicate similarity and also dissimilarity in SM and WB traits at the metabolic level.

In the current study, 15-HETE increased in both SM and WB compared to N. 15-HETE is an eicosanoid, a metabolite of arachidonic acid, and it is involved in inhibiting inflammation response (Zhu and Ran, 2012). Consistently, a previous study by Wu et al. (2024) reported that 14,15-dihydroxyeicosatrienoic acid was increased in SM compared to N, potentially due to inflammatory response in SM. A previous study by Wang et al. (2010) reported that 15-HETE had important roles in inhibiting cell apoptosis and improving cell survival in pulmonary arterial smooth muscle cells of rats. Moreover, Abasht et al. (2016) demonstrated that elevated 15-HETE in WB would be a defensive mechanism to inhibit inflammation due to muscle myopathies. These results indicate that inflammation was induced in both SM and WB. A previous study by Lake et al. (2019) demonstrated that ectopic lipid accumulation is one of the primary metabolic characteristics of WB in broiler chickens. Accordingly, in the current study, several lipid related metabolites including alpha-dimorphecolic acid, 15(S)-HETE, and (±) 18-HEPE were upregulated in SM and WB compared to N. The SM and WB are known to have the greater fat content level compared to normal breast meat (Tasoniero et al., 2020; Liu et al., 2022). The increased 15-HETE could be due to upregulated lipid metabolism and increased fat accumulation in WB and SM compared to N.

Inositol phosphates play important roles in regulating various signal transduction pathways responsible for cell growth and differentiation, cell signaling, apoptosis, DNA repair, RNA export, regeneration of ATP, etc. (Kuksis, 2003). In the current study, D-inositol-4-phosphate decreased in SM and WB compared to N. Reduced D-inositol-4-phosphate in SM and WB indicates problems in cell homeostasis, which could induce muscle myopathies in the breast meat. The WB and SM had 19 and 10 upregulated metabolites related to ABC transporters, respectively, in the current study. ABC transporters have important roles in transporting various substrates and importing nutrients and exporting toxic metabolites in an energy dependent manner (Linton, 2007). The analysis of KEGG classification revealed that differential metabolites related to metabolic pathways and biosynthesis were the greatest among the KEGG pathways in both SM and WB when compared to N. These results suggest that altered cellular metabolism could play a role in the development of SM and WB, but there would be more dramatic changes in cellular metabolism of WB compared to SM.

In the current study, purine metabolism was found to be upregulated in both SM and WB compared to N, with WB showing greater number of differential metabolites related to purine metabolism compared to SM. Consistently, Lake et al. (2022) also demonstrated that purine metabolism was upregulated in the blood of broilers with WB. Purine metabolism, which includes the *de novo* purine biosynthetic pathway, purine salvage pathway, and degradation, is essential not only for DNA and RNA synthesis but also for supplying vital energy and cofactors to promote cell survival and proliferation (Pedley and Benkovic, 2017; Yin et al., 2018). Boerboom et al. (2022) suggested that increased purine metabolism in WB would be associated with increased carbon metabolism and/or decreased utilization of nucleotides. Moreover, in the current study, the differential metabolites associated with nucleotide metabolism were overall upregulated in SM and WB. Potentially, the repair and regeneration process would be induced in SM and WB. Upregulated metabolites related to purine metabolism, particularly uric acid, could be associated with muscle myopathies since uric acid inhibits xanthine oxidase, which plays crucial roles in alleviating diverse health issues including endothelial dysfunction, insulin resistant, hepatic steatosis, and muscle atrophy (Derbre et al., 2012; Miller et al., 2024). However, in the current study, folic acid levels decreased in SM and WB compared to N. Folic acid, which can only be obtained from the diet, is an essential water-soluble vitamin B9 and crucial for cell division and growth by regulating the biosynthesis of nucleic acids and proteins (Ratajczak et al., 2021). In addition, folic acid may regulate the functions and integrity of skeletal muscle cells (Hwang et al., 2019). Liang et al. (2022) demonstrated that supplementation with a high dose of folic acid exhibited beneficial effects on broiler meat production by activating the mammalian target of rapamycin (mTOR) pathway, leading to increased protein deposition in the breast muscle. To our knowledge, there were no studies demonstrating the interaction between folic acid and muscle myopathies in broiler chickens. It would be of interest to investigate the association between the different folic acid levels and muscle myopathies in broiler chickens.

Neoxanthin, which is a kind of carotenoid and xanthophyll that can be found in corns (major feed ingredient in broiler feed), has health promoting effects in animals (Moreno et al., 2016). In the current study, neoxanthin decreased in WB compared to N. Carotenoids as vitamins, can also play important roles as antioxidants in animals (Nogareda et al., 2016). Oxidative stress can be readily induced in broiler chickens due to diverse factors including fast-growth, bacterial and parasitic infection, stimulated immune system, and consumption of mycotoxin (Choi et al., 2020). In the current study, taurine, glutathione, and the reduced form of glutathione were increased in SM and WB compared to N, which indicates that oxidative stress was induced in SM and WB. A previous study by Abasht et al. (2016) demonstrated that the increase in taurine and glutathione could be due to the tissues’ exposure to high levels of oxidants in WB. Based on our current results, induced oxidative stress could be one of the etiologies for SM and WB. Potentially, decreasing the oxidative stress and increasing antioxidants in the feeds could be strategies to reduce the incidence and severity of SM and WB.

In the current study, differential amino acid related metabolites (amino acids, polypeptides, small peptides, and amino acid derivatives) were observed in SM (185) and WB (312) compared to N. Moreover, between SM and WB, there were 211 differential amino acid related metabolites. The analysis of KEGG classification revealed that 20- and 4 differential metabolites associated with biosynthesis of amino acids were observed in the comparisons between WB and N and between SM and N. This result indicates that the WB condition had more impacts on amino acid metabolism compared to the SM. Between WB and N, 111 amino acid related metabolites were downregulated, but 201 amino acids related metabolites were upregulated. Between SM and N, 103 amino acid related metabolites were downregulated, but 82 amino acids related metabolites were upregulated. Overall, these results suggest that amino acid related metabolites in WB were upregulated (201 up and 111 down), but amino acid related metabolites in SM were generally downregulated (82 up and 103 down). Soglia et al. (2019) reported that free amino acids were increased in WB potentially due to myogenesis within the abnormal muscle. Potentially, myogenesis in WB could induce the overproduction of amino acids. However, a previous study by Dalle Zotte et al. (2020) reported that WB had reduced amino acid content and modulated profile of amino acids compared to the N. Our previous study also displayed that the WB had lower crude protein content compared to N, which suggest reduced free amino acids in WB (Choi et al., 2024). This difference could originate from the analytic method. This would be because the untargeted metabolomics analysis does not incorporate the actual concentrations of metabolites. The overall reduction in amino acid related metabolites in SM relative to N is consistent with our previous study showing that there were alterations and reductions in myofibrillar proteins in SM (Tasoniero et al., 2022). Thus, the lack or the modulation of certain amino acids in the breast muscle would be associated with the detachment of muscle myofibers in SM. In addition, aminoacyl-tRNA biosynthesis is an important process for protein synthesis, pairing tRNAs with their corresponding amino acids for decoding mRNA (Gomez and Ibba, 2020). When WB were compared to SM or N, the 13 differential metabolites related to aminoacyl-tRNA biosynthesis were upregulated. This would be consistent with upregulated amino acids related metabolites in WB. The alterations in aminoacyl-tRNA biosynthesis could explain the modulation of amino acids and it could be one of the etiologies in WB.

Nicotinamide adenine dinucleotide (NAD) + hydrogen (H) (NADH) plays a vital role as a cofactor in cellular respiration, participating in both glycolysis and the citric acid cycle by serving as an electron carrier. In the present study, the abundance of NADH was exclusively decreased in SM compared with N. However, significant differences in NADH were not observed between WB and N. This implies that reduced levels of NADH may be the main distinguishing factor in SM compared to WB. This is consistent with the observation by Mazzoni et al. (2020) that the stained area for reduced NADH- tetrazolium reductase (NADH-TR) was significantly smaller in meat with the SM condition, whereas no differences were observed in WS and WB compared with N. These results indicate that there could be severe defects in the energetic metabolism and mitochondrial disorders in SM. High energy demands are implicated in the development of skeletal muscle in fast-growing broilers (Mohammadabadi et al., 2021). These results suggest that malfunctions in energy metabolism can result in detachment of fiber bundles (e.g., SM). A previous study by Wang et al. (2023) showed that the etiologies of WB would be associated with reduced glycogenesis, glycolysis, and energy metabolism. Consistently, in the current study, the compromised carbon metabolism was one of the top 10 differential metabolomic pathways according to KEGG analysis between WB compared to N. However, only 3 differential metabolites were associated with carbon metabolism between SM to N. Whereas SM had lower abundance of NADH compared to N, reduced NADH in SM did not result in a modulation of carbon metabolism, which is a potential etiology to induce WB. More studies are needed 1) to elucidate the association between the deficiency of NADH and the SM condition and 2) to investigate the effects of dietary supplementation of NADH or NADH precursors on the incidence and severity of SM.

It is well known that steroid hormones have important roles in promoting muscle protein synthesis and hypertrophy in skeletal muscles (Dalle Zotte et al., 2020). In the current study, stigmastane-3,6-dione, a natural sterol, was the most decreased differential metabolites in SM and WB compared to N. This result indicates that alterations in steroid hormones could be one of etiologies for both SM and WB. In the present study, there were 10 differential metabolites related to steroid hormone biosynthesis between SM and N; however, there were only 5 between WB and N. Although 21-deoxycortisol, aldosterone hemiacetal, tetrahydrodeoxycorticosterone, androsterone, cortisone, 11 beta-hydroxyandrostenedione, (8S,9S,10S,13S,14S)-17-(2-hydroxyacetyl)-10,13-dimethyl-2,4,5,6,7,8,9,12,14,15,16,17-dodecahydro-1H-cyclopenta[a]phenanthrene-3,11-dione were downregulated, testosterone, 16 alpha-hydroxyestrone, and 2-methoxyestradiol were upregulated in SM. Reduction of steroid hormones potentially induced detachment of fiber bundles (e.g., symptoms of SM) (Carson and Manolagas, 2015). The lack of the movement of broiler chickens in the finisher stage could decrease the biosynthesis of steroid hormones because movement is an important factor to induce synthesis of steroid hormones in skeletal muscles (Sato and Iemitsu, 2015). A previous study by Iresjö et al. (2019) demonstrated that supplementation of amino acids, especially branched chain amino acids (BCAA), induced biosynthesis of estrogen in cultured skeletal muscle cells. In broiler chickens, BCAA play a significant role in muscle development, and the results from our current study may suggest that supplementation of BCAA may reduce the incidence and severity of SM by stimulating biosynthesis of steroid hormones. Further studies are required to verify whether BCAA could regulate biosynthesis of steroid hormones and affect SM in broiler chickens.

In summary, there were similarly and distinctly differential metabolites in SM and WB when compared to N as well as each other as shown in Table 6. Although SM did not induce as substantial alterations in metabolites as WB, there were still significant metabolic changes observed in SM compared with N. These data suggest both shared and unique metabolic alterations in SM and WB, indicating commonalities and differences in their underlying etiologies and meat quality traits. These findings suggest that dietary supplementation of deficient nutrients (e.g., NADH, folic acid, etc.) and modulation of altered pathways in SM and WB could be strategies to decrease the incidence and severity of SM and WB. More focused studies are required to suggest dietary and genetic interventions to decrease incidence and severity of SM and WB in broiler chickens.

**TABLE 6:** Overall description of differential metabolites and pathways between spaghetti meat (SM), wooden breast (WB), and normal (N) fillets.

Distinct in SM		Shared in SM and WB		Distinct in WB	
NADH	↓	Metabolic pathways	↑↓	Amino acids	↑
Amino acids	↓	Biosynthesis of cofactors	↑↓	Aminoacyl-tRNA biosynthesis	↑
Steroid hormone biosynthesis	↓	Stigmastane-3,6-dione	↓	ATP-binding cassette (ABC) transporters	↑
		(±)15-Hydroxyeicosatetraenoic acid (HETE)	↑	Carbon metabolism	↓
		D-inositol-4-phosphate	↓	Neoxanthin	↓
		Amino acids	↑↓		
		Folic acid	↓		
		Oxidative stress	↑		
		Purine metabolism	↑		

## Data Availability

The raw data supporting the conclusions of this article will be made available by the authors, without undue reservation.

## Supplementary material

SUPPLEMENTARY TABLE S1 Differential metabolites between normal (N), spaghetti meat (SM), and wooden breast (WB).

SUPPLEMENTARY TABLE S2 Co-expressed and uniquely expressed metabolites in spaghetti meat (SM) and wooden breast (WB) compared to normal (N).
